# Planning Ahead with Care: A Randomized Clinical Trial of a Video Intervention to Improve Caregiver Knowledge of Severe Dementia and Medical Care Decisions

**DOI:** 10.1002/alz.085405

**Published:** 2025-01-09

**Authors:** Katherine Brandt, Megan Quimby, Bonnie Wong, Lisa M. Quintiliani, Amy Marchesano, Alexa Goldstein, Gent Celaj, Inola Howe, Erin Krahn, Raseeka O’Chander, Samantha Krivensky, Michael K. Paasche‐Orlow, Angelo Volandes, Brad C Dickerson

**Affiliations:** ^1^ Frontotemporal Disorders Unit, Charlestown, MA USA; ^2^ Frontotemporal Disorders Unit, Massachusetts General Hospital, Boston, MA USA; ^3^ Tufts Medical Center, Boston, MA USA; ^4^ Massachusetts General Hospital, Boston, MA USA; ^5^ Frontotemporal Disorders Unit, Massachusetts General Hospital, Charlestown, MA USA

## Abstract

**Background:**

Caregivers of individuals living with dementia frequently make medical decisions for care recipients, often without a clear understanding of those needs. Educational tools are needed to guide caregivers about when and how to raise the topic of advance care planning (ACP).

**Method:**

Caregivers of individuals living with dementia (N = 130) were randomized into control (N = 64) (ACP verbal educational script) or intervention (N = 66) (ACP verbal educational script and video) groups. Both groups completed online surveys and participated in online interviews. The educational intervention was performed at baseline with pre‐ and post‐intervention assessments. Baseline, three‐ and six‐month interviews assessed severe dementia and medical care knowledge quantitatively (true/false questions) and qualitatively (open‐ended questions). Decisional conflict (Decisional Conflict Scale) and decisions for comfort care (open‐ended questions) were also examined.

**Result:**

Across the entire sample, severe dementia and medical care knowledge increased immediately after the educational intervention. Although the groups did not differ in severe dementia or medical care knowledge (all *p* values n.s.), the video intervention group demonstrated increased confidence in knowledge about both topics (*p*<0.05). Post‐intervention, participants’ confidence in severe dementia knowledge and medical care knowledge were higher in the video intervention group vs. control group (*p*<0.05). Chi‐square analysis indicated that decision for comfort care did not differ between groups (*p*>0.196). There was no difference between groups in total decisional conflict score or in any of the 5 subscores, pre‐ or post‐intervention (all *p* values n.s.). However, across all participants there was a decrease in total decisional conflict score and in each subscore pre‐ to post‐intervention (all *p* values < .001).

**Conclusion:**

Caregivers of individuals living with dementia benefit from education about the stage of severe dementia and medical care decisions that may need to be made. Analysis is ongoing for three‐ and six‐month follow‐up sessions.

